# Antibacterial Activity of Biosynthesized Selenium Nanoparticles Using Extracts of *Calendula officinalis* against Potentially Clinical Bacterial Strains

**DOI:** 10.3390/molecules26195929

**Published:** 2021-09-30

**Authors:** José A Hernández-Díaz, Jorge JO Garza-García, Janet M León-Morales, Adalberto Zamudio-Ojeda, Jenny Arratia-Quijada, Gilberto Velázquez-Juárez, Julio C López-Velázquez, Soledad García-Morales

**Affiliations:** 1 Department of Plant Biotechnology, Centro de Investigación y Asistencia en Tecnología y Diseño del Estado de Jalisco, Camino Arenero 1227, 45019 Zapopan, Mexico; cepe_armando@hotmail.com (J.A.H.-D.); jona_jjo@hotmail.com (J.J.G.-G.); jucelopez_al@ciatej.edu.mx (J.C.L.-V.); 2 Department of Plant Biotechnology, CONACYT-Centro de Investigación y Asistencia en Tecnología y Diseño del Estado de Jalisco, Camino Arenero 1227, 45019 Zapopan, Mexico; jmlm21@outlook.es; 3 Centro Universitario de Ciencias Exactas e Ingenierías, Universidad de Guadalajara, Boulevard Gral. Marcelino García Barragán 1421, 44430 Guadalajara, Mexico; nanozam@gmail.com (A.Z.-O.); gilberto.velazquez@academicos.udg.mx (G.V.-J.); 4 Departamento de Ciencias Biomédicas, Centro Universitario de Tonalá, Universidad de Guadalajara, Av. Nuevo Periférico Oriente 555, 45425 Tonalá, Mexico

**Keywords:** selenium nanoparticles, antibacterial activity, green synthesis, biomedical applications

## Abstract

The use of selenium nanoparticles (SeNPs) in the biomedical area has been increasing as an alternative to the growing bacterial resistance to antibiotics. In this research, SeNPs were synthesized by green synthesis using ascorbic acid (AsAc) as a reducing agent and methanolic extract of *Calendula officinalis* L. flowers as a stabilizer. Characterization of SeNPs was performed by UV-vis spectrophotometry, infrared spectrophotometry (FTIR), scanning electron microscopy (SEM), energy dispersive X-ray spectroscopy (EDX), and transmission electron microscopy (TEM) techniques. SeNPs of 40–60 nm and spherical morphologies were obtained. The antibacterial activity of marigold extracts and fractions was evaluated by disk diffusion methodology. The evaluation of SeNPs at different incubation times was performed through the colony-forming unit (CFU) count, in both cases against *Serratia marcescens*, *Enterobacter cloacae*, and *Alcaligenes faecalis* bacteria. Partial antibacterial activity was observed with methanolic extracts of marigold leaves and flowers and total inhibition with SeNPs from 2 h for *S. marcescens*, 1 h for *E. cloacae*, and 30 min for *A. faecalis.* In addition, SeNPs were found to exhibit antioxidant activity. The results indicate that SeNPs present a potentiated effect of both antimicrobial and antioxidant activity compared to the individual use of marigold extracts or sodium selenite (Na_2_SeO_3_). Their application emerges as an alternative for the control of clinical pathogens.

## 1. Introduction

Nanotechnology is an emerging area of science that deals with the study and manipulation of matter at the molecular and atomic levels [1]. With the emergence of nanotechnology, a new industrial revolution was generated that impacted the world, especially in the biomedical area, where the physicochemical and biological properties of nanoparticles (NPs) have been widely used and exploited [2]. It gave rise to nanomedicine, considered a new discipline of medicine, whose main areas of application include diagnostics, imaging, and smart drug delivery systems to increase therapeutic efficiency by selective delivery to specific targets [3], as well as the generation of diverse nanomaterials against pathogenic microorganisms such as fungi and bacteria [4].

In general, one of the fundamental characteristics of NPs is that they have a larger surface area, a property different from those of ionic or metallic elements on a macroscopic scale [5]. Due to this, interest has arisen in using elements such as selenium (Se) to obtain NPs. Se is a fundamental trace element for human health, which has structural and enzymatic functions necessary for the proper functioning of the immune system, counteracting the incidence of viruses such as HIV and maintaining sperm motility [6]. However, it has been reported that inorganic Se presents a high degree of toxicity in the human system when exceeding the recommended dose (0.070 mg Se/day for men and 0.060 mg Se/day for women) [5]. On the contrary, the use of SeNPs reduces the possible damage caused to the organism, representing a new mechanism of transport and nanomedical application [7]. It could be exploited to treat many affections, such as cancer, diabetes, and diseases caused by viruses and bacteria, since it has also been shown to have antioxidant and antimicrobial properties [8].

In this study, methanolic extracts of marigold leaves and flowers and fractions of the flower extracts were obtained and evaluated as antibacterial agents. From these results, the extract that presented the highest antibacterial activity was used to synthesize SeNPs, using AsAc as a reducing agent and the marigold extract as a stabilizer. The evaluation of the reduction process and stability of SeNPs was performed by UV-vis spectroscopy. FTIR determined the functional groups present in the methanolic extract of marigold flowers and SeNPs. SEM and TEM analyzed the size and morphology of SeNPs. The presence of Se in the analyzed NPs was confirmed by EDX spectroscopy. The antibacterial activity of SeNPs was evaluated by counting colony-forming units as a function of different incubation times against three clinically important bacteria, such as *S. marcescens*, *E. cloacae,* and *A. faecalis*. Finally, the antioxidant activity of the methanol extract of flowers and SeNPs was determined.

## 2. Results and Discussion

### 2.1. Antibacterial Activity of Plant Extracts

**Figure 1:**
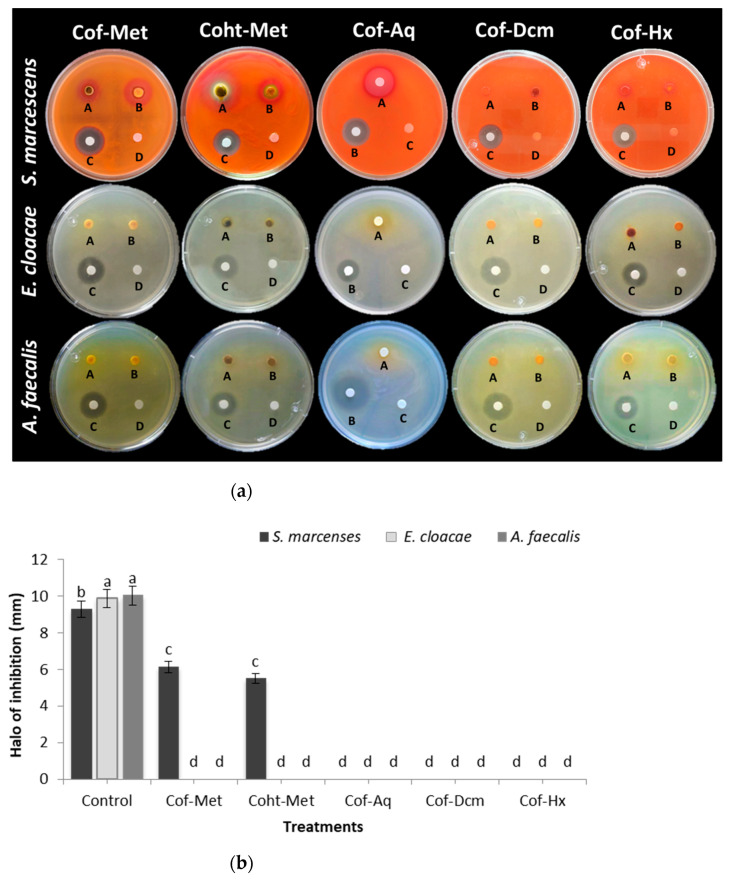
Antibacterial activity of methanol extracts (leaves/stems and flowers) and fractions (hexane, dichloromethane, and aqueous) of marigold flowers. (**a**) Inhibition halos with the different extracts and fractions of marigold on *S. marcenses*, *E. cloacae*, and *A. faecalis*, Cof-Met (A = 300 mg/mL, B = 200 mg/mL, C = Control (*S. marcenses* and *E. cloacae*, gentamicin (Ge) 1 mg/mL, *A. faecalis*, ciprofloxacin (CIP) 1 mg/mL), D = MeOH), Coht-Met (A = 300 mg/mL, B = 200 mg/mL, C = Control, D = MeOH), Cof-Aq (A = 300 mg/mL, B = Control, C = H_2_O), Cof-Dcm (A = 300 mg/mL, B = 200 mg/mL, C = Control, D = AcEt), Cof-Hx (A = 300 mg/mL, B = 200 mg/mL, C = Control, D = AcEt). (**b**) Comparison of means of inhibition halos. Mean values ± SE. Different letters denote statistically significant differences according to Duncan’s test (α = 0.05), *p* < 0.0001. Cof-Met: crude methanolic extract of flowers, Coht-Met: crude methanolic extract of leaves and stems, Cof-Aq: aqueous fraction of flowers, Cof-Dcm: dichloromethane fraction of flowers, Cof-Hx: hexane fraction of flowers.

Two methanolic extracts of leaves/stems and flowers were evaluated for their antibacterial activity against three Gram-negative bacteria (*S. marcescens*, *E. cloacae*, and *A. faecalis*). Three fraction extracts, hexane (Hx), dichloromethane (CH_2_CL_2_), and aqueous, from marigold flowers were tested in the same form. Methanolic extracts of marigold leaves/stems (Coht-Met) and flowers (Cof-Met) exhibited comparable antibacterial activity between them at 300 mg/mL, as shown by the growth inhibition zones (Figure 1a). Comparable results were presented by Efstratiou et al. [9], which demonstrated the antibacterial potential of methanolic extracts of marigold flowers at this same concentration against a large number of bacteria, such as *Bacillus subtilis*, *Escherichia coli*, and *Staphylococcus aureus*. The results of the disk diffusion method indicated that both Coht-Met and Cof-Met had a similar antibacterial effect against *S. marcescens* (Figure 1b). For the case of *E. cloacae* and *A. faecalis*, no inhibitory activity of either methanolic extracts or fractions was found. It could be because the compounds present in the extracts/fractions were not active against the microorganisms used [9].

Both the extracts and the fractions showed a change in medium coloration because of the pigment’s production as carotenoids (astaxanthin, *β*-carotene, zeaxanthin, canthaxanthin, lycopene). The carotenoid’s precursors are two 5C subunits: (a) isopentenyl diphosphate (IPP) and (b) dimethylallyl diphosphate (DMAPP) (isomeric form) through themethylerythritol 4-phosphate (MEP) pathway, as part of the microorganism secondary metabolism. In the specific case of *A. faecalis*, the pigmentation produced by this bacterium can protect against oxidative stressand reinforce bacterial membranes [10]. Based on the results obtained from the evaluation of the antibacterial activity of both the extracts and their fractions, the Cof-Met extract was used to synthesize SeNPs.

### 2.2. Green Synthesis and Characterization of SeNPs

NPs were synthesized by reducing Na_2_SeO_3_ semi-metallic ions using AsAc, inducing the formation of nucleation centers, leading to the formation of SeNPs. To the reaction, marigold flower extract was added as a stabilizing agent. Similar data have been reported showing that marigold extracts serve as a stabilizing agent during the synthesis of NPs [11]. Likewise, the coloration change of the reaction was examined for 0, 3, 9, 15, 30 min, and 24 h, observing the shift from faint yellow to brownish red due to the excitation of the surface plasmon resonance, which indicated the formation of SeNPs. During the formation of SeNPs, similar coloration changes have been reported with green synthesis mediated by *Trichoderma* spp. [12] and AsAc [13].

**Figure 2:**
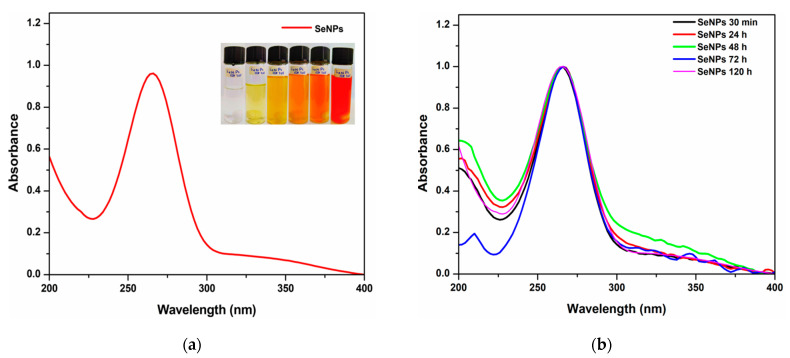
UV-vis spectra of SeNPs. (**a**) Characteristic absorption peak andcoloration change from yellow to red during the synthesis of SeNPs mediated by AsAc and methanolic extract (Cof-Met) at 0, 3, 9, 15, 30 min, and 24 h after the reduction onset. (**b**) Absorption spectra of SeNPs measured at different times showing the stability of SeNPs.

The SeNPs were characterized by the UV-vis technique. The UV-vis spectrum shows an absorption peak at a wavelength of 265 nm, scanning in the range of 200 to 400 nm (Figure 2a). It agrees with Vahdati and Moghadam [14], who report an absorption peak at this wavelength using AsAc for the synthesis of SeNPs. On the other hand, the stability of the SeNPs was verified by measuring the absorbance at different times, finding that the SeNPs stabilized with the marigold extract were stable from 30 min to 120 h after synthesis, suggesting the stabilizing capacity of the extract due to the presence of functional groups such as carboxylic acids and amino groups on the surface of the SeNPs, preventing their agglomeration (Figure 2b) [11]. On the other hand, studies by Fierascu et al. [15] revealed that bioactive compounds such as carotenoids and chlorophylls in the plant extract of marigold flowers are responsible for the stability of the NPs.

**Figure 3:**
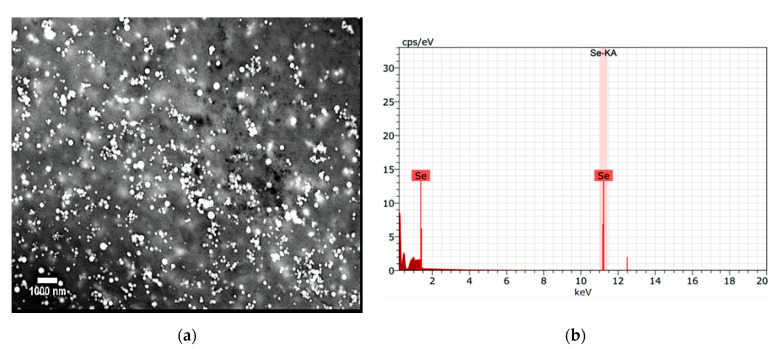
Characterization of SeNPs. (**a**) Micrograph of SeNPs at 34 kx by SEM microscopy. (**b**) EDX spectra showing the presence of Se.

Regarding the morphology and size of the synthesized SeNPs, the SEM technique was performed at 20 kV and 34 kx, using backscattered electrons (Figure 3a), and it was observed that the biosynthesized SeNPs presented a monodisperse spherical morphology with an approximate size of 100 nm. This size obtained by SEM sometimes shows clusters rather than individual SeNPs, making it impractical to identify the boundary between individual NPs. Similar sizes and morphologies of SeNPs have been reported using *Pelargonium zonale* extract [16]. In this regard, Filipovic et al. [17] demonstrated that the shape and size of NPs are decisive parameters that determine the interaction between NPs and the biological systems in which they are used. In turn, EDX analysis confirmed the presence of elemental Se in the analyzed SeNPs sample (Figure 3b). Characteristic absorption peaks of Se were found at 1.37 keV (SeLα peak) and 11.22 keV (SeKα peak), which is in agreement with the information obtained by Dhanjal and Cameotra, who indicated intense spectral peaks for SeKα at 11.22 KeV [18].

**Figure 4:**
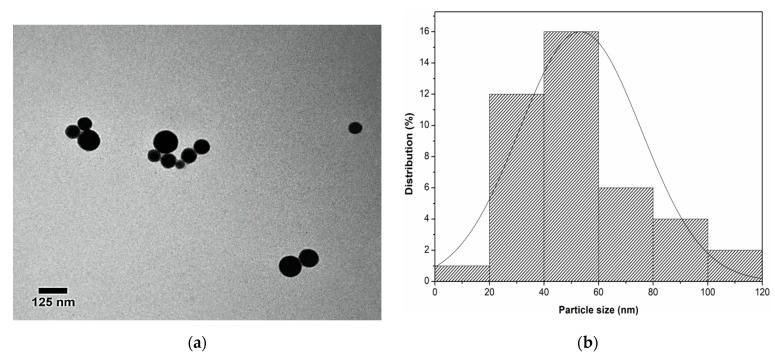
Characterization of SeNPs by TEM microscopy. (**a**) Micrograph of SeNPs taken at 120 kx. (**b**) Size distributionplot of SeNPs.

The TEM technique was used to corroborate the shape and size of the synthesized SeNPs. TEM images were obtained at 80 kV and 120 kx magnification. In the micrographs of SeNPs, obtained by AsAc-mediated green synthesis and Cof-Met extract, a spherical morphology and an average frequency size distribution of 40 to 60 nm in diameter were observed (Figure 4a,b). It has been reported that changes in the morphology and size of NPs may be due to different nucleation and growth mechanisms related to factors such as temperature and reaction time [19]. For example, there are reports that the average size of Co-Fe_2_O_4_ NPs increased with exposure to a reaction temperature above 40 °C, increasing from 9 nm at this temperature to 47 nm at 100 °C [20]. This work found that a maximum temperature of 40 °C for 30 min resulted in the optimal conditions for obtaining SeNPs with a size below 100 nm.

The results obtained from TEM (Figure 4a) agree with the morphology of the SeNPs found with the SEM technique (Figure 3a). However, SeNPs size was larger by SEM because the energy used in this technique is lower than that required by TEM, which decreases the resolution of the micrographs.

**Figure 5:**
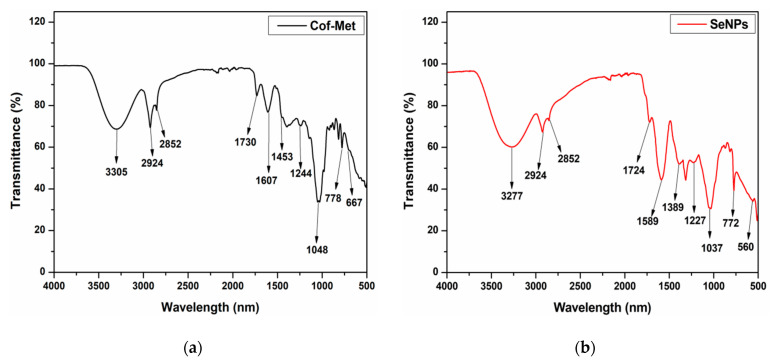
Fourier transform infrared spectroscopy (FTIR) analysis. (**a**)FTIR spectra of Cof-Met extract used for the biosynthesis of SeNPs. (**b**) FTIR spectra of SeNPs.

The infrared spectrum of the Cof-Met extract and SeNPs was measured in the wavelength range of 4000–500 cm^−1^ (Figure 5). The peaks were found to represent the Cof-Met extract’s functional groups, the AsAc, and the interaction with Se. It suggests the ability of marigold extracts to adhere to the surface of NPs as a form of stabilization, as proposed by Olfati et al. [11], who used aqueous extracts of this same plant as a stabilizing agent for NPs. According to the results of the FTIR spectra, it was possible to identify some absorption bands at 3305 cm^−1^ representing the stretching of the hydroxyl groups (-OH) of alcohols and phenols present in the methanolic extract of flowers. In contrast, a shift of this band to 3277 cm^−1^ was observed in the SeNPs. This same behavior was presented during the biosynthesis of SeNPs mediated by the aqueous extract of *Emblica officinalis* fruit, where a band shift from 3382 to 3348 cm^−1^ was reported, indicating the interaction of Se with the -OH group present in alcohols and phenols of the extracts through hydrogen bonds [21,22]. Similarly, bands at 2924 and 2852 cm^−1^ were observed, which correspond to functional groups such as carboxylic acid and C-H of alkane groups, possible biomolecules responsible for stabilizing SeNPs [11,23].

In turn, the spectrum of the Cof-Met extract (Figure 5a) indicated the presence of bands at 1730 and 1607 cm^−1^ attributed to stretching and vibrational bending of the carbonyl (C=O) region and vibrations of soluble protein amides, while for the SeNPs, these bands were shifted to 1724 and 1589 cm^−1^, respectively. According to Rajathi et al. [24], this behavior could indicate a possible interaction of these compounds with Se. Likewise, the Cof-Met extract presented bands at 1453 cm^−1^, corresponding to the C-C, C-O, and C-N stretching of organic compounds, and 1048 cm^−1^ attributable to the C-OH stretching of alcohols [25]. At the same time, it has been reported that bands between 1038 and 1602 cm^−1^ indicate the presence of carbohydrates and proteins [26]. For SeNPs (Figure 5b), the latter band shifted to 1037 cm^−1^, representing the characteristic vibration of Se-O stretching, achieving the formation of SeNPs in the reducing medium with AsAc, as reported by Kannan et al. [27]. On the other hand, in SeNPs, the band at 560 cm^−1^ corresponds to the out-of-plane bending vibrations of the aromatic C-H groups [28], which, according to Stella et al. [29], originate from the stretching vibrations of the metal–oxygen bond. Likewise, the band at 772 cm^−1^ was found in SeNPs, as indicated by Jahdaly et al. [30], andsuggests the binding of SeNPs to the -OH groups present in AsAc, giving rise to Se-O coordination bonds between Se and AsAc. The Se–OH interaction nature could be due to the binding of the SeNPs to the C=O groups of the AsAc oxidation products, forming a layer around the SeNPs to avoid their agglomeration [13].

### 2.3. Antibacterial Activity of SeNPs

Bacterial resistance to antibiotics is one of the significant problems facing medicine. Currently, infection-related problems persist despite the intense efforts made by the scientific community. Given this, many researchers have focused their study on the development of nanostructured inorganic compounds with antimicrobial activity, such as metals, semi-metals, metal oxides, non-metal oxides, and others [31]. Among these compounds, the use of SeNPs represents an alternative to conventional products as they are classified as agents with significant antimicrobial activity, especially when dealing with chronic and nosocomial infections caused by bacteria [17].

The antibacterial activity of SeNPs against three Gram-negative bacteria was evaluated by measuring the growth of bacterial colonies at different incubation times and treatments.

*S. marcescens* was selected because it was initially considered a non-pathogenic bacterium due to its low virulence in healthy populations. However, in the last 30 years, this bacterium has emerged as a nosocomial pathogen causing wound and eye infections, urinary and respiratory tract infections, and causing endocarditis, osteomyelitis, septicemia, and meningitis [32,33]. In several clinical cases of peritoneal dialysis patients, it is considered one of the Gram-negative bacteria with one of the worst diagnoses, causing in some cases the loss of the catheter, the failure of the technique, and the death of the patient [34]. In addition, analyses of *S. marcescens* have shown an increase in antibiotic resistance. Thus, it is often described as a difficult-to-treat bacterium with a high antibiotic resistance profile [32].

**Figure 6:**
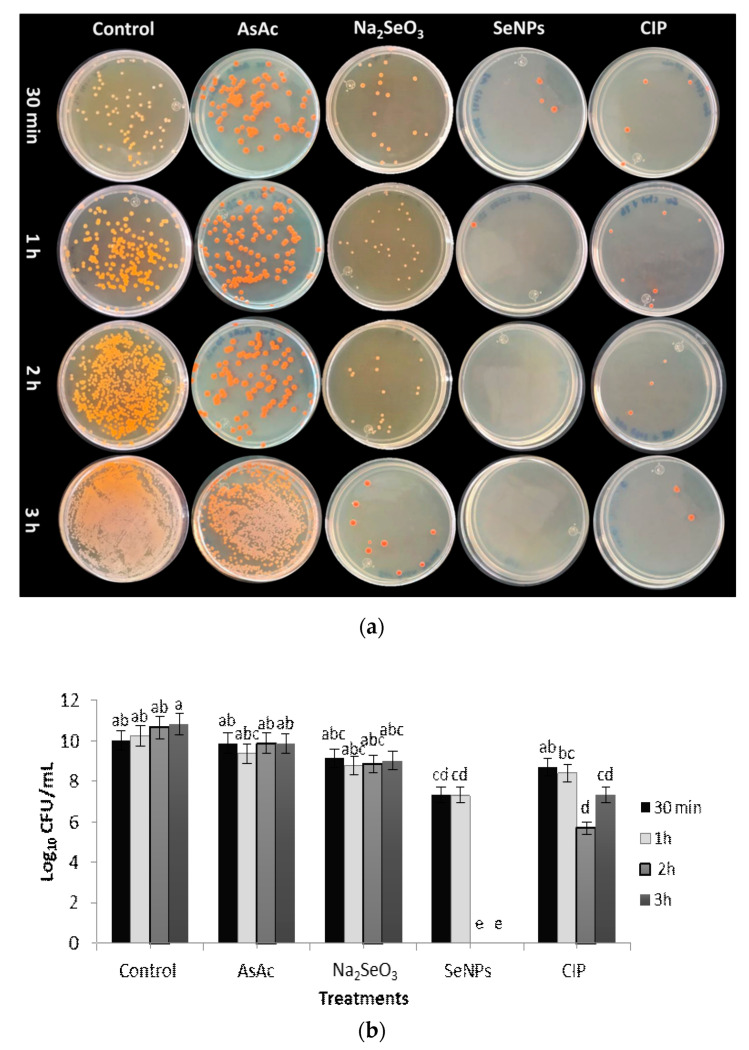
Antibacterial activity of SeNPs against *S. marcescens*. (**a**) Evaluation of antibacterial activity under different treatments and incubation times. (**b**) Effect of SeNPs on *S. marcescens* colony formation. Mean values ± SE. Different letters denote statistically significant differences according to Duncan’s test (α = 0.05), *p* < 0.0001.

The application of SeNPs against *S. marcescens* showed inhibitory results similar to the control (CIP) after 1 h of incubation. At the same time, it was determined that after 2 h, the treatment with SeNPs completely inhibited the growth of the bacterium (Figure 6). These results are in agreement with those presented by Menon et al. [35], where the use of SeNPs (size > 100 nm) showed significant inhibition on the same bacteria after 5 h of incubation. Based on these results, it can be established that the size of SeNPs plays a relevant role in antibacterial activity, as described by Jeong et al. [36], who indicated that to achieve a more significant antibacterial effect, NPs should be smaller than 100 nm in size. The higher antibacterial activity found in this work is due, in part, to the fact that SeNPs have a size between 40 and 60 nm with a larger contact surface area, and could more easily cross the cell wall and bacterial membrane, inducing cell lysis, interfering with ATP synthesis and affecting cell division, leading to bacterial cell death [37].

These results highlight the importance of SeNPs against non-*Pseudomonas* Gram-negative bacteria [38]. It is mainly due to the significant increase in the risk of hospitalization caused by *S. marcescens* compared to other bacteria [39]. One of the mechanisms involved in the pathogenicity of this bacterium in humans is the formation of biofilms, polymeric substances that provide protection against antibiotics and host defense mechanisms. In this sense, it has been reported that NPs can penetrate through biofilms’ water channels, causing their disruption [40]. Inhibiting or eliminating the formation of biofilms could be an effective way to reduce their pathogenicity, which could counteract nosocomial infections caused by this bacterium. For the specific case of SeNPs, anti-biofilm efficacy against pathogens such as *Bacillus cereus*, *Enterococcus faecalis*, *S. aureus*, and *E. coli* have been reported in other studies [41]. Therefore, SeNPs could be used as an alternative for eliminating bacterial biofilms while decreasing the possibility of antibiotic resistance developed by bacteria.

**Figure 7:**
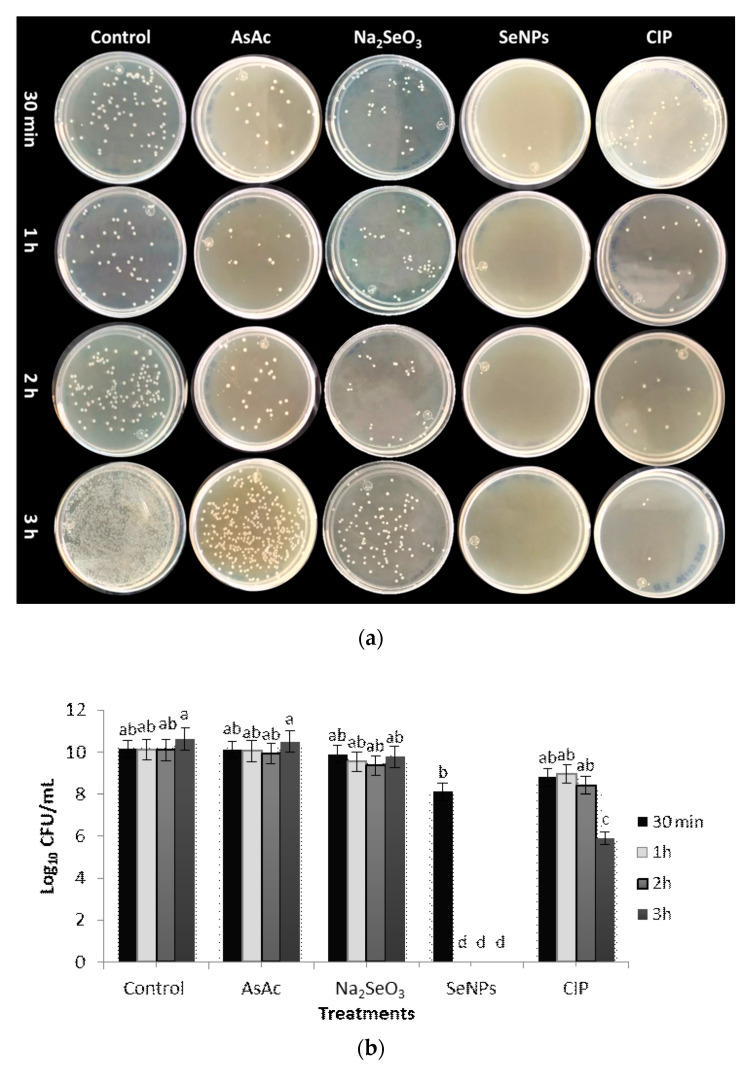
Antibacterial activity of SeNPs against *E. cloacae*. (**a**) Evaluation of antibacterial activity at different treatments and incubation times. (**b**) Effect of SeNPs on colony formation. Mean values ± SE. Different letters denote statistically significant differences according to Duncan’s test (α = 0.05), *p* < 0.0001.

The evaluation of the antibacterial activity against *E. cloacae* was similar to that found in *S. marcescens*. Partial inhibition of bacterial growth was observed at 30 min and total inhibition at 1 h after applying the SeNPs. These results were statistically superior to those obtained by the CIP, which required 3 h to present a significant inhibition in the formation of bacterial colonies vs. the control (Figure 7a,b). This effect can be attributed to the cell-specific presence of lipopolysaccharide-lipoprotein complexes in the cell wall of Gram-negative bacteria and the presence of transport pumps, which allow bacteria to regulate their internal environment by removing toxic substances such as antimicrobial agents. For the specific case of *E. cloacae*, the EmmdR pump has been reported, while for *S. marcescens*, the SdeAB pump. In both cases, the presence of these pumps induces resistance in antibiotics such as CIP [42]. These mechanisms prevent antibiotics from reaching the intracellular sites necessary to exert their antibacterial function [43]. It is suggested that SeNPs present different mechanisms of action, such as the generation of reactive oxygen species (ROS), disruption of the bacterial cell wall, and inhibition of protein and DNA synthesis [44].

In addition, one of the advantages of using NPs as an antibacterial agent is their ability to act through multiple mechanisms, resulting in the inability of pathogens to acquire resistance to them [17,44]. In Na_2_SeO_3_, a decrease in CFU was found after 1 and 2 h of incubation. After 3 h, the trend changed, increasing CFU due to a possible bacterial Se adaptation. So far, it has been reported that bacteria of the genus *Pseudomonas* [45], *E. cloacae* [46], and *Azoarcus* sp. [47] can synthesize SeNPs from Na_2_SeO_3_ as a mechanism to reduce Se toxicity internally.

*A. faecalis* is considered as an emerging drug-resistant pathogen, causing opportunistic infections in humans. In most cases, infection generated by *A. faecalis* is complicated to treat because it is highly resistant to several antibiotics, such as anti-pseudomonal penicillin, cephalosporins, carbapenemics, aminoglycosides, and quinolones [48]. Currently, cases of *A. faecalis* infection have been identified, including cystitis, skin and soft tissue infection, pneumonia, bacteremia, meningitis, endocarditis, eye infection, peritonitis, and infectious diarrhea [49].

**Figure 8:**
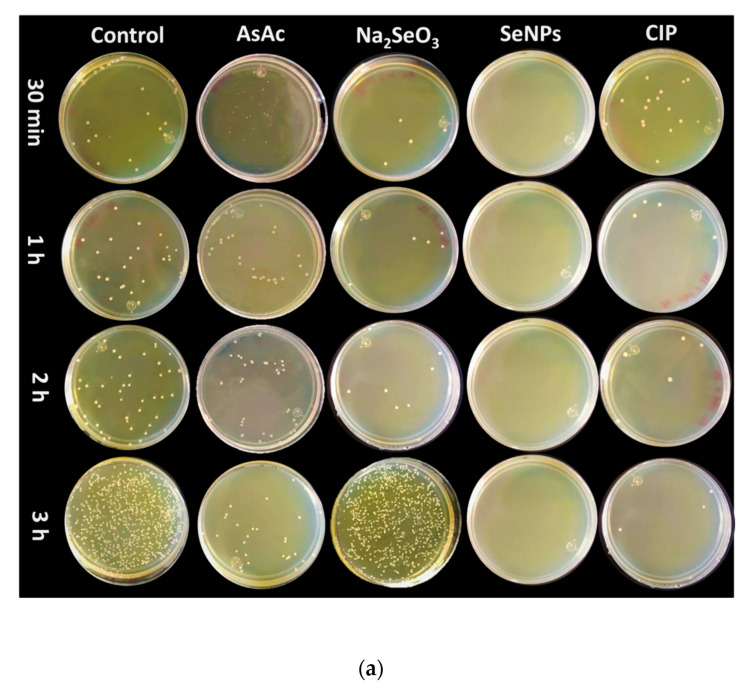

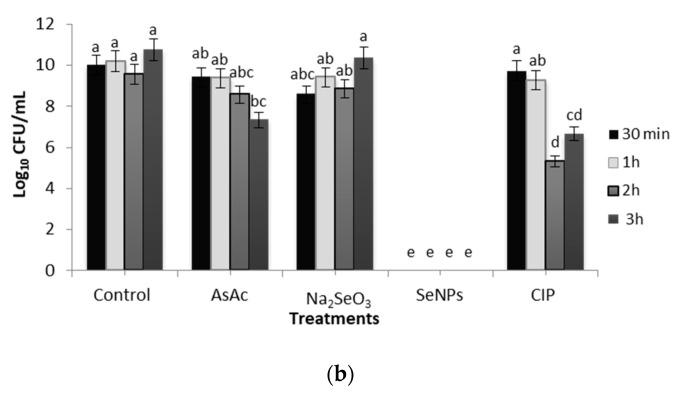
Antibacterial activity of SeNPs against *A. faecalis*. (**a**) Evaluation of antibacterial activity under different treatments and incubation times. (**b**) Effect of SeNPs on bacterial colony formation. Mean values ± SE. Different letters denote statistically significant differences according to Duncan’s test (α = 0.05), *p* < 0.0001.

In this research, it was possible to observe an inhibition of total growth from the first incubation time (30 min) using SeNPs (Figure 8a,b). It could be due to the content of bioactive compounds in the Cof-Met extracts present on the surface of the SeNPs, capable of damaging the signaling receptors necessary to carry out quorum sensing (QS) [50], a chemical process of cell-to-cell communication used by bacteria such as *A. faecalis* to regulate their virulence and the formation of biofilms [51,52]. This QS inhibition has been reported using SeNPs biosynthesized with AsAc, with the average size of 85 nm, where the inhibition of the biosynthesis of crucial QS compounds such as violacein (80%) was demonstrated, suggesting the disruption of the QS signal [53]. On the other hand, CIP showed significant results after 1 h vs. the control.

Likewise, the results obtained with Na_2_SeO_3_ reflect a similar tendency of adaptation to Se as observed in *S. marcescens* and *E. cloacae*. For this case, the ability of *A. faecalis* to reduce Na_2_SeO_3_ to SeNPs has been reported, establishing as apossible reduction mechanisms the action of cellular reductases and reducing compounds whose production is related to the growth phase of the bacterium [54]. Additionally, the more significant inhibitory activity of AsAc on *A. faecalis* was observed, which could be related to a decrease in bacterial viability, as obtained by Gnarpe et al. [55], who through treatments with AsAc against *A. faecalis* were able to decrease viable cell counts. This effect could be due, in part, to the fact that bacterial growth is usually slow in acidic media. Therefore, acidifying agents such as AsAc are often used to decrease pH. At the same time, AsAc can interfere with the activity of signaling molecules in the QS such as AI-2, affecting cell-to-cell signaling in bacteria [56].

On the other hand, SeNPs assays against *A. faecalis* are limited.One of the few reports is by Hegerova et al. [57], who used SeNPs functionalized with carboxymethylcellulose, chemically synthesized and with an average size of 50–150 nm, and they found highly effective activity against *A. faecalis* with a growth inhibition halo of 17 mm in diameter. Whereas, in this study, SeNPs obtained by biological synthesis with AsAc and marigold extracts, with a size of 40–60 nm, were found to inhibit the growth of *A. faecalis* completely. According to these results, it could be considered that this bacterium presents high sensitivity to this type of antibacterial agent. In addition, it was observed that both methods of synthesis (chemical and biological) of SeNPs were effective in inhibiting bacterial growth and that the size of the NPs was not determinant in the antibacterial activity of these nanomaterials.

This work addresses the use of SeNPs as an antibacterial agent against nosocomial pathogenic bacteria, two of them of emerging type (*S. marcescens* and *A. faecalis*), where there is a low number of studies on the use of SeNPs as an alternative for their control, compared to the broad knowledge generated against other bacteria such as *S. aureus* or *P. aeruginosa*. Since a high antibacterial potential of the synthesized and characterized SeNPs was obtained, the subsequent studies have to be focused on performing biocompatibility assays in cell line models in vitro by different cytotoxicity assays

### 2.4. Antioxidant Activity of SeNPs

SeNPs biological activity is closely related to their antioxidant properties, and can be expressed as the relative measure of substance ability to prevent or retard substrate oxidation. SeNPs exhibit antioxidant effects by modulating the generation of ROS and reactive nitrogen species (RNS). These characteristics are exploited in biomedical applications, where SeNPs exhibit anti-inflammatory, anti-diabetic, antiviral, and fertility-enhancing properties [58]. Antioxidant compounds act through different mechanisms, which depend on the nature of the substance itself and its ability to interact with the medium in which it is dissolved [59]. Some chemical forms of Se, either organic (Se-Met and Se-Cys) or inorganic (SeO_4_^2−^, SeO_3_^2−^, Se^2−^), have been reported to exhibit antioxidant activity [60]. In this aspect, it has been recognized that SeNPs have a better antioxidant capacity than the available inorganic forms of Se, and simultaneously the risk of toxicity is reduced [61]. As reported by Vera et al. [62], it describes the ability of SeNPs to scavenge free radicals (OH^•^ and O_2_^•−^).

**Figure 9:**
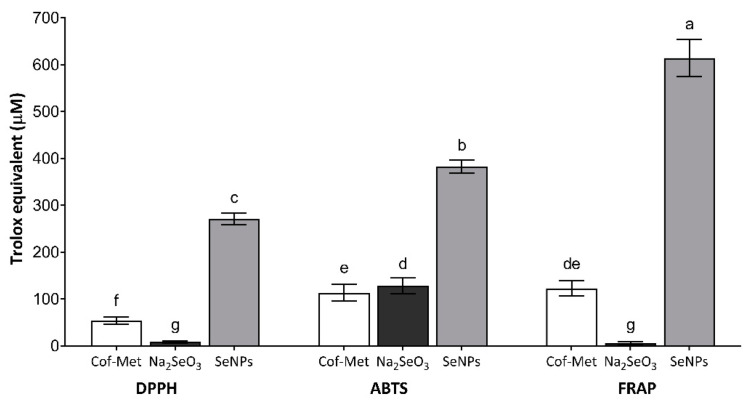
Antioxidant activity of SeNPs determined by DPPH (2,2-diphenyl-1-picrylhydrazyl), ABTS (2,2-azino-bis-3-ethylbenzothiazoline-6-sulphonic acid), and FRAP (Ferric reducing antioxidant power) assays. Cof-Met extract at the concentration used for the synthesis of SeNPs. Na_2_SeO_3_ solution at the concentration used for SeNPs synthesis. Mean values ± SE. Different letters denote statistically significant differences according to Duncan’s test (α = 0.05), *p* < 0.0001.

On the other hand, it is known that various marigold extracts can present compounds with biological activities, including antioxidant activity [63]. This work evaluated the antioxidant activity of the Cof-Met extract, Na_2_SeO_3_, and SeNPs by three different techniques (DPPH, ABTS, and FRAP) (Figure 9). From the antioxidant activity of the Cof-Met extract, values of 54.2 ± 3.1 µM Trolox equivalent by DPPH, 79.7 ± 3.1 µM Trolox equivalent by ABTS, and 79.7 ± 6.7 µM Trolox equivalent by FRAP were obtained. Interestingly, the antioxidant activity of the Na_2_SeO_3_ solution, at the concentration used for the synthesis of the NPs, only presented antioxidant activity with the ABTS technique (128.3 ± 6.6 µM Trolox equivalent), with no values with DPPH or FRAP. The conditions of the FRAP (acid pH) and DPPH (methanol) methods could affect the inhibition kinetics of the radical, generating a result ofundetected activity for Na_2_SeO_3_ in both assays. However, the ABTS technique is known to have less aggressive conditions (methanolic extract and less acidic pH) that facilitate the detection of specific antioxidant molecules.

The SeNPs presented high values of antioxidant activity compared to the controls (Na_2_SeO_3_ and Cof-Met extract). Likewise, significant differences were obtained between the three techniques used, with values of 271.0 ± 5.1, 382.5 ± 5.6, and 614.2 ± 16.0 µM Trolox equivalent for DPPH, ABTS, and FRAP, respectively. In the case of the antioxidant activity of SeNPs determined by FRAP, the value in µM of Trolox equivalent was 1.6 times higher than the ABTS method and 2.3 times higher than the DPPH method (Figure 9). These data may be striking, but they correspond to a phenomenon related to the high sensitivity of this system to determine, with more responsiveness, various antioxidant compounds whose potential is lower than the pair Fe^3+^–Fe^2+^, which is employed in the assembly of this technique [64]. It is important to note that the antioxidant activity of SeNPs could be affected by the size of the NPs. Studies have reported it based on the analysis of ROS production in human umbilical vein endothelial cells. It was argued that SeNPs biosynthesized with *Pantoea agglomerans* and L-cysteine, with sizes between 30 and 300 nm, showed a particle size-dependent antioxidant capacity [65].

The results obtained from the measurement of antioxidant activity confirm that the possible mechanism of action, mediated by SeNPs, is more oriented towards an electron transfer (ET) reaction mechanism rather than a hydrogen atom transfer (HAT) mechanism. These results are in agreement with the studies performed by Zhai et al. [66] on SeNPs. Additionally, it is noted that the mechanisms of antioxidant activity present the opposite effect (there ishigher activity in DPPH vs. FRAP) when Se is in organic form, as reported by Alafeef et al. [67]. Finally, the results obtained present similar trends to those presented by other authors when comparing between Na_2_SeO_3_ controls vs. SeNPs, since SeNPs possess higher antioxidant activity than the inorganic Se source used for the synthesis of NPs [68].

## 3. Materials and Methods

### 3.1. Plant Material Production

The marigold plant material (leaves/stems and flowers) was obtained from the Costa Orange variety (Balls, Chicago, IL, USA). Seeds were sown in germination trays with commercial substrate and maintained at 25 °C with a photoperiod of 14 h light and 10 h dark, watering three times a week and applying Hoagland nutrient solution 25% once a week. Thirty-day-old seedlings were transplanted into pots with commercial substrate and placed in a greenhouse with average conditions of 18.9 °C, 70.4% relative humidity, and a photoperiod of 11 h light and 13 h dark. The plants were irrigated four times per week with running water and applied to Hoagland nutrient solution 100% once per week.

### 3.2. Preparation of Plant Extracts and Their Fractions

The extracts were prepared using marigold flowers as well as stems with leaves. The plant material obtained was frozen at −80 °C in an ultra-freezer (Forma 900-series, ThermoFisher, Waltham, MA, USA). Subsequently, the plant tissue was freeze-dried in a freeze-dryer (FreeZone, Labconco, Kansas City, MO, USA). Afterward, the plant samples were pulverized in an industrial mill (MF10BS1, IkaWerke, Wilmington, DE, USA) until a fine particle size was obtained. For flowers, extraction was performed with methanol (MeOH) (1:20 *w/v*) assisted with sonication, using an ultrasonic bath of 100 W maximum rated output power and 42 kHz frequency (2510R-DTH, Branson Ultrasonics, Danbury, USA) for three periods of 15 min at constant temperature (25 °C). For stems/leaves, they were macerated in MeOH (1:10 *w/v*) for 24h periods in triplicate. Finally, the solvent was evaporated under reduced pressure using a rotary evaporator (R-100, Buchi, Flawil, Switzerland) at a bath temperature of 40 °C. The extracts obtained were stored at 4 °C.

For fractionation of the methanolic flower extract, the first 37.0g of the extract was dissolved in 370 mL of MeOH (1:10 *w/v*). Subsequently, distilled water (3:2, *v/v*) was added. The resulting aqueous suspension was successively partitioned with hexane (twice; 1:1 *v/v*) and dichloromethane (1:1 *v/v*). Finally, the aqueous fraction was the resultant of both fractionations. The solvents were evaporated at 40 °C in a rotary evaporator (R-100, Buchi, Flawil, Switzerland). The methanolic extracts of flowers, leaves/stems, and the resulting fractions were frozen at −80 °C and then freeze-dried (FreeZone, Labconco, Kansas City, MO, USA) for 5 days.

### 3.3. Obtaining and Characterization of Bacterial Strains

Three strains of Gram-negative bacteria isolated from Phaseolus vulgaris seeds (*S. marcescens* and *E. cloacae*) and *P. lunatus* seeds (*A. faecalis*) were used to test both fractions’ antibacterial activity of methanolic extracts of leaves/stems, flowers, and SeNPs. Morphological identification of the isolated bacterial strains was performed by Gram staining. For molecular identification, DNA extraction and amplification of the 16S rRNA fragment of ribosomal DNA was performed by polymerase chain reaction (PCR) in a thermal cycler (C1000 touch, Biorad, Hercules, CA, USA). Universal primers 27F (5′-AGAGTTTGATCCTGGCTCAG-3′) and 1492R (5′-GGTTACCTTGTTACGACTT-3′) were used [69]. The PCR product obtained was purified using a commercial kit (Wizard^®^ SV Gel and PCR Clean-Up System, Promega, Madison, WI, USA), and the purified product was sent for sequencing (Macrogen Inc, Seul, South Korea). The sequences obtained were compared with the sequences deposited in the GenBank genomic database, and a comparison of multiple alignments was performed using the BLAST tool available at http://www.ncbinlmnih.gov/, accessed on 14 September 2021. The consensus sequence of each strain was obtained and identified as *S. marcescens* (MK411566.1, 99.93% identity), *E. cloacae* (MT509937.1, 99.70% identity), and *A. faecalis* (CP033861.1, 99.29% identity). Subsequently, the nucleotide consensus sequence was deposited in GenBank with the accession numbers MZ913727 (*S. marcescens*), MZ914398 (*E. cloacae*), and OK067254 (*A. faecalis*).

### 3.4. Evaluation of the Antibacterial Activity of Plant Extracts and Their Fractions

*S. marcescens*, *E. cloacae*, and *A. faecalis* bacteria were incubated in LB medium (Sigma-Aldrich, St. Louis, MO, USA) at 30 °C in a digital shaking incubator (LSI-3016R, Lab-Tech, Namyangju, Korea) for 24 h.

Bacterial growth was adjusted to an optical density at 600 nm (OD600), using a spectrometer (Biophotometer, Eppendorf, Hamburg, Germany) to analyze antibacterial activity from marigold extracts and their fractions. Evaluation of antibacterial activity was carried out by the Kirby–Bauer disk diffusion method [70], using methanolic extracts of flowers (Cof-Met) and leaves/stems (Coht-Met), as well as the hexane (Cof-Hx), dichloromethane (Cof-Dcm),and aqueous (Cof-Aq) fractions. All treatments were evaluated at 200 and 300 mg/mL, except Cof-Aq (300 mg/mL). The positive control consisted of ciprofloxacin (CIP) 1 mg/mL for *A. faecalis* and gentamicin (Ge) 1 mg/mL for *S. marcescens* and *E. cloacae*. MeOH, water (H_2_O), or ethyl acetate (AcEt) were included as negative controls. Petri dishes with nutrient agar were inoculated with 100 µL of bacterial growth at OD_600_, previously mixed in 4 mL of 50% nutrient agar. Once the Petri dishes were inoculated, filter paper discs (6 mm) impregnated with 20 µL of the different treatments were placed. Petri dishes were incubated at 30 °C in a natural convection incubator (DBO-13, Prendo, Puebla, Mexico) for 24 h. Finally, the inhibition halo was measured. Each test was performed in triplicate with six replicates.

### 3.5. SeNPs Synthesis

The colloidal solution of SeNPs was obtained by the green synthesis method by reduction of sodium selenite (Na_2_SeO_3_) (Sigma-Aldrich, St. Louis, MO, USA), using 10 mL (10 mM), to which 3.5 mL of ascorbic acid (AsAc) (40 mM) and 120 µL of methanolic extract of marigold flowers (Cof-Met) (100 mg/mL) were added. The reaction was carried out under magnetic stirring at 1150 rpm and 40°C for 30 min. The reaction was completed after 24 h in darknesswithout magnetic stirring at 4 °C.

### 3.6. SeNPs Characterization

The determination of the absorption spectrum and the stability of the SeNPs were obtained using a UV-vis spectrophotometer (Genesis 10S UV-vis, Thermo Scientific, Carlsbad, CA, USA) in the wavelength range from 200 to 400 nm with a path length of 1 cm. The size and shape of the SeNPs were determined by SEM (MIRA 3 LMU, TESCAN, Brno, Czech Republic) at 20 kV and TEM (JSM-1010, JEOL, Tokyo, Japan) at 80 kV. A descriptive statistical analysis was performed on the size distribution of SeNPs from three independent assays. A mean of 53.30 nm, median of 48.72 nm, mode of 48.72 nm, standard error of 3.49, as well as Kurtosis coefficient (0.1011) and skewness coefficient (0.8387) close to zero were obtained. From these data, the normal distribution plot (Gaussian distribution) of the SeNPs size was plotted.Surface microanalysis microprobe EDX spectroscopy (Quantax EDS, Bruker, Billerica, MA, USA) was used to detect the elemental composition of the SeNPs. The functional groups present in the extracts and the resulting SeNPs were determined by FTIR spectrophotometry (iS10 FTIR, Thermo Scientific, Waltham, MA, USA).

### 3.7. Evaluation of the Antibacterial Activity of SeNPs

From axenic cultures, a suspension of each bacterial strain was prepared from an individual colony that was inoculated in a tube with 5 mL of nutrient broth (Difco, Detroit, USA) for *S. marcescens* and *E. cloacae*, while *A. faecalis* was inoculated in 5 mL of LB broth (Sigma-Aldrich, St. Louis, MO, USA). The cultures were incubated at 37 °C for 24 h in an incubator (Incucell IC 55, MMM Group, Munich, Germany). After the incubation time had elapsed, an inoculum was prepared with peptonized water (Difco, Detroit, USA) by adjusting to a concentration of 0.5 on the McFarland scale using a densitometer (Densimat, Biomérieux, Lyon, France), equivalent to 1 × 10^8^ CFU/mL. Five inoculums of each strain were prepared according to the treatments to be evaluated.

For the evaluation of antibacterial activity, each of *S. marcescens*, *E. cloacae,* and *A. faecalis* inoculums were treated with SeNPs (7.3 mM), AsAc (10.27 mM), Na_2_SeO_3_ (7.3 mM), and CIP (1 mg/mL). An untreated inoculum was considered as a negative control. Inoculums with each of the treatments were incubated for 30, 60, 120, and 180 min at 37 °C. After the incubation time, a 100 µL aliquot of each treatment was taken and plated in duplicate on nutrient agar Petri dishes using the surface spreading technique with a Drigalsky loop. Petri dishes were incubated at 37 °C in a natural convection incubator (Incucell IC 55, MMM group, Munich, Germany) for 24 h. For all treatments, dilutions were performed (7 serial dilutions 1/10), and from the 1 × 10^−7^ dilution, an aliquot of 100 µL was taken and plated as previously described. Finally, countable bacterial colonies (25–250 CFU) were recorded, and the results were reported as the average CFU/mL of three independent assays.

### 3.8. Antioxidant Activity Evaluation of SeNPs

The ABTS Assay (ABTS^+^) was conducted as described by Re et al. [71] at 750 nm, and Flores et al. [72]. A solution of ABTS was prepared by diluting 96 mg of ABTS with 25 mL of water; subsequently, 26 mg of potassium persulfate wasadded and mixed by stirring. This solution was stored for 16 h in the dark. At the end of this period, the solution was adjusted to an optical density of 0.7 at 754 nm. A standard curve (0–400 µg/mL) was prepared with Trolox. The reaction was carried out in a microplate, mixing 20 µL of the sample with 280 µL of ABTS. Measurements were performed in triplicate at 754 nm for 15 min.

The Ferrous reducing antioxidant power (FRAP) assay of samples was evaluated by the method of Oyaizu [73]. The Fe^2+^ was monitored by measuring the formation of Perl’s Prussian blue at 700 nm. Briefly, the FRAP reagent was prepared by mixing acetate buffer (300 mM, pH 3.6), a solution of 10 mM 2,4,6-Tripyridyl-s-triazine(TPTZ) in 40 mM HCl, and 20 mM FeCl^3+^ at 10:1:1 (*v/v/v*). The reagent (3.4 μL) and sample solutions (100 μL) were added to each well and mixed thoroughly. The absorbance was taken at 593 nm after 15 min. A standard curve was prepared using different concentrations of Trolox.

The DPPH assay was performed according to Desmarchelier et al.’s [74] protocol. In brief, a 1.6 mM solution of DPPH was prepared. The calibration curve was performed in the range of 0–400 µg/mL using Trolox as a reference. Twenty µL of the samples were added toa micro-plate intriplicate, and 280 µL of the DPPH solution was added subsequently, and readings were measured at 515 nm after 15 min of reaction.

All results of antioxidant activities were expressed as μM Trolox equivalent/mL solution.

### 3.9. Statistical Analysis

A two-way analysis of variance (ANOVA) was performed and the difference between mean values was obtained with the procedures of SAS 9.1 statistical software (SAS Institute, Cary NC, USA), using Duncan’s multiple range test with a significance level of *p* < 0.05.

## 4. Conclusions

Methanolic extracts of marigold leaves/stems and flowers and their fractions were evaluated, and antibacterial activity was obtained using Coht-Met and Cof-Met extracts against *S. marcescens*.

SeNPs were obtained by green synthesis using AsAc and Cof-Met extract. Their characterization by UV-vis, FTIR, SEM, EDX, and TEM showed NPs with a spherical shape and a 40–60 nm size range. The antibacterial activity of the extract, AsAc, and Na_2_SeO_3_ was enhanced by producing the SeNPs, which significantly inhibited the growth of *S. marcescens*, *E. cloacae*, and *A. faecalis* bacterial strains. The results of the antibacterial assays demonstrated that the SeNPs presented better antibacterial activity, at different incubation times, compared to the antibiotic CIP. The application of Na_2_SeO_3_ and AsAc showed an inhibitory effect on the growth of bacterial colonies. However, this activity was lower than that obtained with SeNPs. The importance of this study lies in the possibility that SeNPs could be an effective alternative for the control of these bacterial strains, whichare associated with hospital infections, are considered of clinical and epidemiological importance, and are classified as resistant to antibiotics. Additionally, it was found that SeNPs present higher antioxidant activity than Cof-Met extract and Na_2_SeO_3_, which could be exploited for the treatment of bacterial disease affections together with their antibacterial activity. Further research is needed to understand the mechanism of action of SeNPs on bacteria, the modes of delivery to the target sites, and the possible toxic effects on living organisms.

## Sample Availability

Samples of the bacterial strains are available from the authors.
